# Clearing the air: improving smoke-free policy compliance at the national oncology hospital in Armenia

**DOI:** 10.1186/1471-2407-14-943

**Published:** 2014-12-13

**Authors:** Narine K Movsisyan, Varduhi Petrosyan, Arusyak Harutyunyan, Diana Petrosyan, Frances Stillman

**Affiliations:** School of Public Health, American University of Armenia, Yerevan, Armenia; Institute for Global Tobacco Control, Bloomberg School of Public Health, Johns Hopkins University, Baltimore, MD USA

**Keywords:** Smoke-free policy, Smoke-free hospital, Secondhand smoke (SHS), Indoor tobacco smoke pollution, Policy compliance, Armenia, Transition economies

## Abstract

**Background:**

Smoke-free policies shown to reduce population exposure to secondhand smoke (SHS) are the norm in hospitals in many countries around the world. Armenia, a transition economy in the South Caucasus, has one of the highest male smoking rates in the European region. Although smoking in healthcare facilities has been banned since 2005, compliance with this ban has been poor due to lack of implementation and enforcement mechanisms and social acceptability of smoking. The study aimed to develop and test a model intervention to address the lack of compliance with the *de jure* smoking ban. The national oncology hospital was chosen as the intervention site.

**Methods:**

This study used employee surveys and objective measurements of respirable particles (PM_2.5_) and air nicotine as markers of indoor air pollution before and after the intervention. The intervention developed in partnership with the hospital staff included an awareness campaign on SHS hazards, creation of no-smoking environment and building institutional capacity through training of nursing personnel on basics of tobacco control. The survey analysis included paired t-test and McNemar’s test. The log-transformed air nicotine and PM_2.5_ data were analyzed using paired t-test.

**Results:**

The survey showed significant improvement in the perceived quality of indoor air, reduced worksite exposure to SHS and increased employees’ awareness of the smoke-free policy. The number of employees reporting compliance with the hospital smoke-free policy increased from 36.0% to 71.9% (p < 0.001). The overall indoor PM_2.5_ concentration decreased from 222 μg/m^3^ GM (95% CI = 216-229) to 112 μg/m^3^ GM (95% CI = 99-127). The overall air nicotine level reduced from 0.59 μg/ m^3^ GM (95% CI = 0.38-0.91) to 0.48 μg/ m^3^ GM (95% CI = 0.25-0.93).

**Conclusions:**

The three-faceted intervention developed and implemented in partnership with the hospital administration and staff was effective in reducing worksite SHS exposure in the hospital. This model can facilitate a tangible improvement in compliance with smoke-free policies as the first step toward a smoke-free hospital and serve as a model for similar settings in transition countries such Armenia that have failed to implement the adopted smoke-free policies.

**Electronic supplementary material:**

The online version of this article (doi:10.1186/1471-2407-14-943) contains supplementary material, which is available to authorized users.

## Background

As part of a comprehensive tobacco control strategy, smoke-free policies have been shown to reduce exposure to secondhand smoke, increase quitting rates and reduce overall smoking prevalence [1, 2]. There is less resistance to establishing smoke-free hospitals because of their mission of prompting health. Hospitals can serve an important access points to deliver smoking cessation advice [3] and healthcare professionals can be important role models to promote smoke-free norms and behaviors [4, 5].

Clearing hospitals from tobacco smoke is still underway around the world. Most of the evidence on successful smoke-free policy interventions is based on the US or other high-income countries where a major shift occurred based on evidence of the harmful health effects of secondhand smoke (SHS) [1, 6–8]. However, little data are available in transitional countries where resources are scarce to effectively implement health policies protecting the public from SHS exposure. Furthermore, more research needs to focus on what can be done when an institution has a policy but fails to adequately implement or enforce it leading to poor compliance and occurrence of smoking where it is formally prohibited.

Armenia, a transition economy in the South Caucasus, has one of the highest male smoking rates in the European region (55.1% male; 3.7% female) and was the first in the post-soviet region to join the world treaty on tobacco control, the Framework Convention on Tobacco Control in 2004 [9, 10] The Armenian tobacco control law enacted in early 2005 prohibits smoking in educational, cultural and healthcare facilities. However, enforcement and compliance with the ban has been insufficient and a multi-country study in 2007 found high levels of tobacco indoor air pollution in public places in Yerevan, Armenia [11, 12]. Thus, though being in place, the national anti-smoking policies are not properly implemented. This study aimed to develop, implement and test a model intervention to improve the compliance with the adopted (*de jure*) but not being actually followed smoke-free policy in the national oncology hospital in Yerevan, Armenia.

## Methods

### Setting

The study was conducted in a 500-bed tertiary referral hospital located in the capital city Yerevan that provides comprehensive cancer care. The hospital that had a few unsuccessful attempts to go smoke-free in recent years was chosen as an intervention site.

### Intervention

The research team developed and implemented a model smoke-free intervention in fall 2009 in close cooperation with the hospital leadership. The first step of the intervention included formation of a coordinating committee in charge of the smoke-free intervention implementation in the hospital. Led by the hospital deputy director, this committee included the head nurse, a young physician experienced in tobacco control programs, the coordinator of the state tobacco control program and representatives of the research team. To inform and enrich the intervention development process the study team explored the employees’ smoking-related attitudes and perceived barriers for implementation of smoke-free policy in the hospital through focus group discussions (FGDs) with nurses and physicians [13]. In addition, the research team conducted structured observations to understand in which specific indoor locations smoking occurs in the hospital. The results of the preliminary research were shared with the coordinating committee to help with development of specific intervention steps. To finalize the plan for the smoke-free intervention in the hospital, the research team also reviewed a few international case studies [14–21].

The intervention included the following three facets:

1. Information campaign about the hazards of SHS exposure and benefits of having a smoke-free hospital
  The information campaign targeted hospital staff, patients and visitors and used a variety of channels. The senior administration informed the hospital personnel about the smoke-free policy to be established and the intervention steps at regular staff meetings. The patients and visitors were informed about the policy through: a) large signs about the hospital smoke-free policy placed at the entrance to the hospital, b) no-smoking signs referencing the national tobacco control law and informing about penalties in case of violations posted on all floors of the hospital, c) leaflets with information on health hazards of smoking and SHS, benefits of smoke-free hospitals and the national ban of smoking in healthcare facilities, and c) verbal notifications about the smoke-free policy by hospital nurses.
2. Establishing “no-smoking” environment.
  All the ashtrays were removed from the hospital and were replaced with garbage cans with a no-smoking sign.
3. Building institutional capacity to maintain no-smoking environment.
  Nurse-managers of all clinical departments participated in two-day “Training of Trainers” sessions. The trainings aimed to extend nurses’ knowledge on dangers of smoking and SHS exposure and their understanding of the benefits of smoke-free policy in the hospital, and to introduce the basic approaches in smoking cessation counseling. The nurse-managers received packages of relevant materials to use during the trainings of department nurses. A shorter training on basics of tobacco control was also organized for nurse aides to enhance their role in implementing smoke-free policy in the hospital. These trainings helped to build employees’ support for implementation of smoke-free policy.
  The official launch of the smoke-free intervention took place on the occasion of the National No Tobacco Day (October 12) and was marked by a well-covered press conference to emphasize the importance of becoming a smoke-free hospital and gain support and attention from the community at large.

### Study design

To evaluate the effectiveness of the smoke-free hospital intervention, the study used an employee survey along with objective measurements of indoor tobacco smoke pollution taken before and two months after the intervention (panel evaluation design). The study team assessed indoor air pollution using 1) passive sampling of vapor-phase air nicotine and 2) active monitoring of concentration of respirable particles ≤2.5 μg/m^3^ (fine particular matter, PM_2.5_) in the hospital building.

### Survey

The survey assessed practices, attitudes and beliefs of the hospital physicians, nurses and other staff members on smoking, worksite smoking exposure, and non-smoking policies. All available clinical, administrative and ancillary staff members (full and part time) were eligible for the study. The trained interviewers contacted first the heads of all clinical and administrative departments and then available staff members to explain the study aims and procedures and to ask for verbal consent. The consented employees were handed a coded questionnaire to be returned in a sealed envelope. The team made several visits to cover all shifts in the departments. The study team used a self-administered questionnaire developed by the Institute for Global Tobacco Control team at Johns Hopkins University [22] that was adapted for this study. The 42-item survey questionnaire included standardized questions on socio-demographic variables and smoking status, behavior, and attitudes toward smoke-free policy, perceived indoor air quality and frequency of observed indoor smoking.

### Objective measurements

#### PM_2.5_ measurements

The research team carried out PM_2.5_ measurements in the hospital in April and December 2009 at three purposively selected locations: the waiting area of the surgery department, the administration floor and the cafeteria, assuming their higher occupancy by visitors and staff. The PM_2.5_ concentrations were measured using a TSI SidePak AM510 Personal Aerosol Monitor [23]. The measurements were carried out for 30 minutes, unobtrusively (not to interfere with the natural behavior of hospital employees and visitors) using a convenient shoulder bag with a tube’s end protruded outside the bag. The SidePak was pre-calibrated (calibration factor of 1.0) and the data logging interval was set to 1 minute. All data were measured by the same device.

#### Air nicotine passive sampling

The study team used passive samplers of vapor-phase air nicotine to measure air nicotine concentrations inside the hospital [24]. In addition to the three locations where PM_2.5_ measurements were taken, air nicotine samplers were placed in a few other areas of the main building. Twenty four air nicotine samplers (including two blank and two duplicate monitors for quality control) were placed before (April 2009) and after (December 2009) the intervention, each for 7 days. The study team applied the standard protocol for the air monitors’ labeling, placement, collection and storage [25]. After dropping the blank and duplicate samplers and two others that were damaged or lost, 18 pairs of devices were eligible for the analysis. The air samplers were analyzed at the Exposure Assessment Facility at the Johns Hopkins Bloomberg School of Public Health (JHSPH) for nicotine content analysis by gas chromatography technique. The limit of detection was set at 0.0085 μg/m^3^.

### Ethical approval

The Institutional Review Boards of the American University of Armenia and the JHSPH reviewed and approved the study protocols.

### Data analysis

The research team entered and cleaned the survey data with SPSS11for Windows and analyzed using STATA/SE12 statistical packages. We analyzed the survey participants’ socio-demographic baseline characteristics using chi-square test for categorical and independent t-test and Anova for continuous variables. Self-reported smoking behavior, beliefs and attitudes before and after the intervention were compared using paired t-test for continuous variables and McNemar’s test for categorical variables.

The study team also analyzed PM_2.5_ and air nicotine objective measurements data. Because of a skewed distribution of the data, we computed medians, interquartile ranges (IQRs) and geometric means (GM) to describe PM_2.5_ and air nicotine concentrations inside the hospital. Besides, Wilcoxon signed rank sum test was conducted to compare air nicotine medians before and after the intervention and paired t-test was performed on log-transformed air nicotine data. Additionally, we estimated percent difference in air nicotine before and after the intervention on log-transformed data.

## Results

### Survey

#### Survey response rate

In total, 295 employees out of 565 (52.0%) filled the questionnaire at baseline and 246 at follow up (16.9% were lost to follow up and 1 respondent did not fill the baseline questionnaire). No significant differences were found between those lost to follow up and those included in the analysis in terms of age, gender, smoking status and occupation.

#### Survey participants’ baseline characteristics

The survey participants’ mean age at baseline was 44.25 years (sd = 12.04); the majority were women (81.4%) and non-smokers (75.5%). Nurses and physicians comprised 40.5% and 33.8% of the sample, correspondingly. Majority (70.9%) of the study participants at baseline reported never smoking, 17.3% were current smokers and 7.2% ex-smokers. Smoking prevalence differed significantly across the occupation and gender, but not age.

Nearly half (46.5%) of male employees were current smokers as opposed to 11.5% of women (p < 0.001). Male employees smoked more cigarettes as compared to female during working hours (9.6 vs. 2.9, p < 0.01), as well as per day (20.3 vs. 9.9, p < 0.05) (Table 1).

**Table 1:** **Survey respondents’ age and smoking behavior by gender**

	Male	Female	p-value
Age (yrs), mean ± sd	43.37 ± 13.58	44.85 ± 11.65	0.47*
Smoking status % (N)			
Current smoker	46.51(20)	11.48(21)	<0.001**
Ex-smoker	20.93(9)	4.37(8)	
Never smoked	32.56(14)	84.15(154)	
Smoking duration (yrs), mean ± sd	17.82 ± 11.48	16.28 ± 10.77	0.62*
Cigarettes/day, mean ± sd	20.33 ± 15.50	9.93 ± 8.73	0.032*
Cigarettes/day at work, mean ± sd	9.58 ± 8.03	2.94 ± 5.26	0.008*
Quit attempts in 30 days, % (N)	27.78(5)	40.00(6)	0.46**

*independent t-test.

**chi-square test.

Smoking rates were the highest among physicians compared to nurses and ancillary staff (34.2% vs. 8.8% and 10.7%, correspondingly) (Table 2). The duration of smoking (years) and the number of monthly quit attempts within the last month did not differ across occupation and gender (Tables 1 and 2).

**Table 2:** **Survey respondents’ age and smoking behavior by occupation**

	Nurses	Physicians	Non-clinical staff	p-value
Age (yrs), mean ± sd	41.51 ± 10.89	43.08 ± 12.43	51.39 ± 10.56	<0.001*
Smoking status% (N)				<0.001**
Current smoker	8.89(8)	34.18(27)	10.53(6)	
Ex-smoker	1.11(1)	16.46(13)	5.26(3)	
Never smoked	90.0(81)	49.37(39)	84.21(48)	
Smoking duration (yrs), mean ± sd	15.67 ± 8.73	17.39 ± 10.37	17.29 ± 17.5	0.92*
Cigarettes/day, mean ± sd	6.67 ± 7.26	17.50 ± 15.24	20.00 ± 7.07	0.19*
Cigarettes/day at work, mean ± sd (N)	1.00 ± 2.65	7.75 ± 7.99	9.0 ± 7.62	<0.09*
Quit attempts in 30 days, % (N)	16.67(1)	34.78(8)	50.00(2)	0.53**

*one-way Anova.

**chi-square test.

#### Indoor SHS exposure

Hospital employees reported significant improvement in perceived indoor air quality related to tobacco smoke at follow up. The proportion of respondents who assessed it as good or fair increased from 69.5% to 83.6% (p < 0.001). There was also a significant reduction in observing smoking inside the hospital building, including cafeteria, patient lounges, corridors, and stairwells, but not in physicians’ offices (Table 3).

**Table 3:** **Indoor air quality, awareness of the worksite smoke-free policy and indoor smoking behavior before and after the intervention**

Question	Before	After	p-value*
	% (N)	%(N)	
The air quality (tobacco smoke level) in your building is:			
Good/Fair	69.5(162)	83.6(188)	<0.001
Poor	30.5(71)	16.4(37)	
How often do you smell tobacco while you are at work?			
Frequently	53.2(124)	41.8(95)	0.36
Infrequently/Never	46.8(113)	58.2(142)	
Does the hospital have any policy against smoking in the buildings?			
“Yes” answers	75.2(174)	96.4(213)	<0.001
Are the official policies about smoking in the building followed?			
“Yes, it is followed” answers	36.0(82)	71.9(161)	<0.001
In the past 30 days, have you seen people smoking in the following areas?			
“Yes” answers			
Cafeteria	39.6(88)	25.8(54)	<0.001
Offices	57.7(131)	39.6(86)	0.42
Corridors	77.5(179)	53.9(119)	<0.001
Stairwells	80.2(186)	49.6(109)	<0.001
Lounges in patient care areas	33.5(76)	19.3(41)	<0.001
Restrooms	23.3(52)	14.9(31)	<0.001
Outside the building	97.4(226)	95.9(212)	<0.001

*McNemar test.

#### Smoke-free policy awareness

Employees’ awareness of the smoke-free policy improved significantly from 37.7% at baseline to 63.4% at follow up (p < 0.001) (Table 3). Moreover, the number of respondents who reported that the hospital smoke-free policy was observed increased from 36.0% to 71.9% (p < 0.001).

#### Smoking behavior

Survey respondents reported less cigarette smoking and more quitting attempts at follow up than at baseline. Thus, the number of cigarettes smoked daily and during work hours decreased from 15.8 (10.8- 20.8) and 6.5 (3.9- 9.2) to 14.1 (9.6-18.7) and 5.7 (3.3- 8.0), correspondingly. At the same time, 40.0% of smokers recalled a quitting attempt within the last month at follow up as compared to 33.3% at baseline. However, these changes in respondents’ smoking behavior were not statistically significant.

### Objective measurements

#### PM_2.5_ data analysis

**Figure 1:**
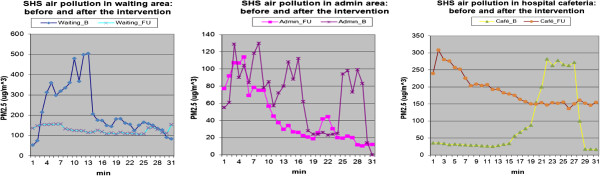
**PM2.5 concentrations before and after the intervention in the hospital.**

The overall indoor PM_2.5_ concentration decreased from 222 μg/m^3^ GM (95% CI = 216-229 μg/m^3^) to 112 μg/m^3^ GM (95% CI = 99-127 μg/m^3^). The paired t-test using log-transformed data showed that changes of PM_2.5_ concentration over time in all three locations were statistically significant, including the decrease in waiting (p = 0.03) and administrative areas (p < 0.001) and the increase in cafeteria (p < 0.001). Figure 1 graphically presents the real-time PM_2.5_ flows in three locations at baseline and follow up.

#### Air nicotine analysis

Aggregated air nicotine level decreased by 18.8% at follow up, from 0.59 μg/m^3^ GM (95% CI = 0.38-0.91) to 0.48 μg/m^3^ GM (95% CI = 0.25-0.93) (see Additional file 1). A reduction in geometric mean values of air nicotine was observed in every location except the doctors’ offices and stairwells; however, these differences were not statistically significant. We found the greatest reduction in the air nicotine GMs in administrative offices, cafeteria and patient lounges, followed by waiting areas (50.98%; 34.76%; 31.58% and 19.50%, accordingly). On the contrary, the levels of air nicotine increased in doctors’ offices and stairwells.

## Discussion

While a number of studies examined implementation of smoke-free hospital policies in different parts of the world [15, 19, 21, 26, 27], none of them were carried out in a transition economy such as Armenia and only a few used objective measurements of indoor air quality. The Armenian legislation prohibits smoking in healthcare institutions; however, this policy has not been sufficiently adhered since its enactment in 2005 [12]. Failure to implement and enforce SHS policies undermines the intent to create a safe and healthy environment. It also builds skepticism towards the occurrence of a meaningful change, as social cognitive theory suggests, due to interaction between person’s past experience, environment, and behavior [28]. Such situations, where a policy formally adopted on the national level is not actually adhered to in particular setting(s), are not a rare case in emerging and re-emerging economies, for example, in China [29]. The goal of our study was to develop and test a model intervention to improve the compliance with *de jure* smoking ban in hospitals in Armenia. Our study proved the intervention to be successful in significantly reducing indoor smoking at the hospital though we did not reach 100% smoke-free. However, the intervention was a good start for improving the compliance with the smoke-free policy as required by the national legislation and it could be scaled up to other hospitals in Armenia. This model can be applied also in neighboring countries with a similar issue of poor compliance with smoke-free policies.

We have identified several barriers to successful implementation of smoke-free policy in hospitals, including high prevalence of smoking among the hospital physicians and their reluctance to accept their role as opinion leaders related to smoking ban [13]. Therefore, the implementation of a smoke-free intervention in the study setting did require a careful planning to address these barriers and not to stigmatize smoking employees. The essential part of this intervention was identifying the lead person who had the authority and willingness to support and lead the effort on part of the hospital administration. The intervention did not require much financial resources; but the leadership and commitment of the hospital’s top administration was crucial.

Our findings suggested significant improvements in employees’ awareness of the smoke-free policy and the indoor air quality after the intervention. These findings from the employee survey were confirmed, to a certain extent, by the objective measurements of air nicotine and PM_2.5_ pollution. We observed reduction of air nicotine in all indoor locations except doctors’ offices and stairwells. The PM_2.5_ levels decreased in the waiting and administrative areas but increased in the cafeteria. The air nicotine in this particular study was more informative as a proxy marker of indoor air pollution because the data were cumulative for seven days while PM_2.5_ measurements were carried for 30 minutes. Therefore, both the survey and objective data suggest that smoking went down in most public areas and increased in physicians’ offices, i.e. that smoking shifted from public areas to less visible places. This could be a good accomplishment for the initial stage of establishing smoke-free policies. Future interventions would need to target smoking in physician’s offices and other less visible areas, such as stairwells. Installation of smoke detectors could complement the educational approach in addressing the “hidden” smoking.

The intervention did not reduce the smoking rates among the hospital employees. However, the smokers reported smoking fewer cigarettes at work and per day and more smokers recalled a quitting attempt in the past 30 days at follow up than at baseline.

The short-term evaluation of the intervention showed positive effects. However, without a proper follow up and leadership these effects may diminish over time [26, 30]. The international experience suggests that when smoke-free policy becomes a requirement in hospital accreditation process as in the US or an adopted code of practice as in the European Smoke Free Hospitals Network, this may substantially help to sustain efforts toward clearing up the smoke in hospitals [26, 31]. In this study, the intervention focused mainly on the hospital staff and did not target patients and their caretakers. Future interventions could include smoking cessation programs for employees and smoking cessation counseling to patients during hospitalization.

This study was implemented at one facility limiting the generalizability of the findings. In addition, the study findings might be affected by seasonal variations in indoor smoking because the objective measurements were conducted in April (baseline) and December (follow up).

## Conclusions

Based on the study findings, we suggest that the three-faceted intervention developed and implemented in a partnership with the hospital administration and staff was effective in reducing worksite SHS exposure in the hospital. This model can facilitate a tangible improvement in compliance with the smoke-free hospital policy as a first step toward a smoke-free hospital and serve as a model for similar settings in transition countries such Armenia that have failed to implement the adopted smoke-free policies.

## Electronic supplementary material

Additional file 1: **Air nicotine concentrations before and after the intervention in the hospital.** (PDF 50 KB)
