# Supplementary material for: Clearing the air: improving smoke-free policy compliance at the national oncology hospital in Armenia
Source: BMC Cancer. 2014 Dec 13;14:943. doi: 10.1186/1471-2407-14-943 (PMC4320561; doi:10.1186/1471-2407-14-943)
Supplement: Supplementary file 1 — Additional file 1: Air nicotine concentrations before and after the intervention in the hospital. (PDF 50 KB) [file 12885_2014_5170_MOESM1_ESM.pdf]

Additional file 1. Air nicotine concentrations before and after the intervention in the hospital

Location	Number of samplers	GM (95% CI) µg/m^3			
		Before	After	p-value*	% reduction
Overall	18	0.59<br>(0.38-0.91)	0.48<br>(0.25-0.93)	0.19	18.84
Waiting area	3	0.82<br>(0.23-2.92)	0.66<br>(0.14-3.03)	0.21	19.51
Patient lounges	4	0.19<br>(0.09- 0.41)	0.13<br>(0.03- 0.61)	0.27	31.58
Stairwells	2	0.96<br>(0.27-3.39)	1.15<br>(0.7-18.96)	0.66	-19.79
Cafeteria	2	1.64<br>(0.29- 9.24)	1.07<br>(0.76-1.49)	**0.08**	34.76
Doctors' offices	3	0.59<br>(0.20-1.69)	0.78<br>(0.01-97.24)	0.60	-32.20
Admin areas	2	0.51<br>(0.21-1.28)	0.25<br>(1.95e-07-318348.7)	0.31	50.98

* paired t-test on log-transformed data
